# Incident gout and chronic Kidney Disease: healthcare utilization and survival

**DOI:** 10.1186/s41927-019-0060-0

**Published:** 2019-03-19

**Authors:** Dena H. Jaffe, Alyssa B. Klein, Arriel Benis, Natalia M. Flores, Hagit Gabay, Robert Morlock, Dana Y. Teltsch, Jonathan Chapnick, Yair Molad, Shmuel M. Giveon, Becca Feldman, Maya Leventer-Roberts

**Affiliations:** 1 Kantar Health, Ariel Sharon St 4, 52511 Ramat-Gan, Israel; 2 AstraZeneca, Medical Evidence and Observational Research Centre, 200 Orchard Ridge Drive, Gaithersburg, MD USA; 3 Clalit Research Institute, Zamenhoff 42, Floor – 1, 6435331 Tel Aviv, Israel; 4 Kantar Health, Foster City, CA USA; 5 YourCareChoice, Ann Arbor, MI USA; 6 Evidera, Waltham, MA USA; 7 Kantar Health, Horsham, PA USA; 8 Beilinson Hospital, Rabin Medical Center and Sackler Faculty of Medicine, Tel Aviv University, Petach Tikva, Israel

**Keywords:** Gout, Chronic kidney disease, Healthcare utilization, Survival

## Abstract

**Background:**

Uncontrolled gout can cause significant joint and organ damage and has been associated with impairments in quality of life and high economic cost. Gout has also been associated with other comorbid diseases, such as chronic kidney disease. The current study explored if healthcare resource utilization (HRU) and survival differs between patients with incident gout in the presence or absence of chronic kidney disease (CKD).

**Methods:**

Clalit Health Services (CHS) data were used to conduct a retrospective population-based cohort study of incident gout between 1/1/2006–31/12/2009. Incident cases of gout were identified and stratified by CKD status and by age group (< 55 and 55+ years). CKD status was defined as a pre-existing diagnosis of chronic kidney disease, chronic renal failure, kidney transplantation, or dialysis at index date. Demographic and clinical characteristics, as well as healthcare resource use, were reported.

**Results:**

A total of 12,940 incident adult gout patients, with (*n* = 8286) and without (*n* = 4654) CKD, were followed for 55,206 person-years. Higher rates of HRU were observed for gout patients with CKD than without. Total annual hospital admissions for patients with gout and CKD were at least 3 times higher for adults < 55 (mean = 0.51 vs 0.13) and approximately 1.5 times higher for adults 55+ (mean = 0.46 vs 0.29) without CKD. Healthcare utilization rates from year 1 to year 5 remained similar for gout patients < 55 years irrespective of CKD status, however varied according to healthcare utilization by CKD status for gout patients 55+ years. The 5-year all-cause mortality was higher among those with CKD compared to those without CKD for both age groups (HR_< 55 years_ = 1.65; 95% CI 1.01–2.71; HR_55+ years_ = 1.50; 95% CI 1.37–1.65).

**Conclusions:**

The current study suggests important differences exist in patient characteristics and outcomes among patients with gout and CKD. Healthcare utilization differed between sub-populations, age and comorbidities, over the study period and the 5-year mortality risk was higher for gout patients with CKD, regardless of age. Future work should explore factors associated with these outcomes and barriers to gout control in order to enhance patient management among this high-risk subgroup.

## Background

Gout is a common, chronic inflammatory disease associated with high serum uric acid (sUA) levels (i.e., hyperuricemia) and characterized by recurrent arthritis attacks induced by monosodium urate crystal deposition throughout the body. Uncontrolled gout can cause significant joint damage, tophaceous deposits, organ damage and comorbidity, as well as impairment in quality of life and substantial economic costs [1–5].

There are clear evidence-based guidelines showing that treating to target levels of sUA, typically using urate-lowering therapy (ULT), will reduce and even remove the burden of this chronic disease from the patient and society [6–10]. The American College of Rheumatology (ACR) and European League Against Rheumatism (EULAR) recommend an sUA target (‘control’) level of < 6 mg/dl in the majority of clinical cases, while the British Society for Rheumatology recommends even stricter guidelines for those with tophaceous gout of sUA target levels < 5 mg/dl [6, 7, 10]. Furthermore, guidelines recommend that gout patients receive urate-lowering therapy (ULT) after diagnosis and routine monitoring, although several studies show that gout patients are poorly managed and that suboptimal treatment of gout is common in clinical practice with few patients undergoing regular sUA testing, poor adherence and compliance to ULT [11–16]. In a study from Germany and the United Kingdom between 2000 and 2005 and with an average follow-up time of about 2 years, over 63% of patients received gout treatment (> 89% with allopurinol), but only 9–14% of patients with gout performed at least one sUA test in the 3.5–5-year follow-up period [11]. Additionally, in a recent study using the cross-sectional US National Health and Nutrition Examination Surveys (NHANES) more than two-thirds of individuals with gout had sUA levels above target and less than half of those treated with ULT reached control levels [17].

Among the many barriers to adequate care and control of this disease is the presence of concurrent kidney disease [18–22]. Approximately 20 to 40% of patients with gout have moderate to end-stage chronic kidney disease (CKD) [20–22]. According to one study assessing gout quality of care, 26% of gout patients with renal failure received inappropriate dosing of allopurinol [13, 23]. Both chronic kidney disease and gout are adversely affected by hyperuricemia and require adequate control to minimize adverse events, comorbidities and mortality [24]. However, despite the clear benefits of proactive care and sUA control, choice and dosing of appropriate drug therapies for these patients are persistent challenges to the healthcare professional [7, 14, 25, 26].

Studies have shown that patients with gout have higher HRU than the general population and that the burden to the healthcare system is increasing [4, 27–31]. In the United States, direct costs of gout were estimated as high as $18,362 per capita, gout-specific costs reaching $6179 per person, and an annual estimated total burden of > $6 billion [4, 31]. Studies show the increased health and cost burden of comorbidities and age on this relationship with regard to HRU [4, 31]. However, the specific contribution of each factor, such as CKD, which is highly prevalent among gout patients, is not fully understood [31].

The objective of this study is to determine if healthcare utilization and survival differs between patients with incident gout in the presence or absence of CKD. We will describe healthcare utilization trajectory during the first 5 years of care for patients with gout with and without CKD, respectively, to provide crucial insights into the health outcomes and disease management of the comorbid patient.

## Methods

### Study database

Clalit Health Services (CHS) is the largest health care payer/provider in Israel, with about 4,217,000 insured citizens that provides care to all ages including > 60% of adults older than 65 years of age in Israel. The system is characterized by extremely low annual member turnover of < 1% [32]. Since 1998, with increasing comprehensiveness, CHS’s information is kept in a central computerized data warehouse that includes integrated demographic data, clinical diagnoses (based on hospital discharge diagnoses, primary care physician diagnoses, and specialist outpatient clinic diagnoses), laboratory data results, medical procedures, and medications (including date of prescription and quantity and time of medication dispensed). Death records, including date of death from the Israel Central Bureau of Statistics, were linked to the Clalit population using the unique identification number for all Israeli residents. The need for consent was waived by the Helsinki Ethics Committee of the CHS (no. 037/2015).

### Study population

This is a retrospective cohort study of newly diagnosed gout between 1/1/2006–31/12/2009 and followed for a 5-year period. For example, patients identified on 1/1/2006 were followed for 5 years through 31/12/2010 and patients identified on 31/12/2009 were followed through 30/12/2014. Follow-up data were included for the partial year the patient left the health plan or died.

**Fig. 1:**
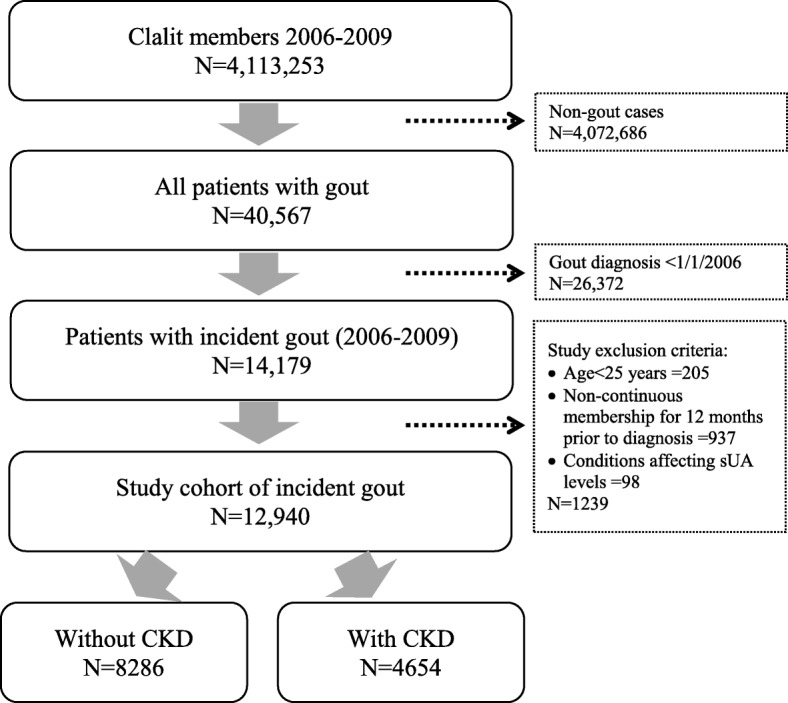
Cohort identification

Included were patients with continuous enrollment in Clalit for 1 year prior to date of diagnosis (index date). Patients had to be at least 25 years old as of index date. Adults 18–24 years were excluded because the majority was serving in the Israeli military where they receive full healthcare coverage. The following criteria developed in other electronic health record (EHR) studies [33–35] to identify incident cases of gout were used (Fig. 1):

1. International Classification of Diseases 9th version (ICD-9) codes 274 diagnosis from at least one rheumatologist visit;
2. ICD-9 274 diagnosis or free-text diagnosis of ‘gout’ from at least two community diagnoses at least 30 days apart between and either
  - the purchase of at least two gout-related prescription medications (allopurinol, probenecid, colchicine, or sulfinpyrazone) at least 30 days apart with the first within 6 months prior to or any time after the first community diagnosis or
  - two sUA test results > 6 mg/dL with the first within 6 months prior to or any time after the first community diagnosis at least 30 days apart;
3. ICD-9 274 diagnosis from at least one hospital admission diagnosis;
4. Clalit Health Services internal chronic diagnosis registry, based on ICD-9 diagnostic codes, diagnostic free text, procedures and test results [36]; and
5. Clalit Health Services physician-determined diagnosis given a ‘permanent’ status in the patient’s medical record, based on ICD-9 diagnostic codes.

Subject with at least one of these were considered to have gout. The earliest diagnosis was considered the index date, and patients were required to have 12 months without any indication of gout (baseline period) to be considered newly diagnosed (incident cases). Gout patients who had documentation of at least one of the above criteria prior to the start of study eligibility were excluded. Patients whose free-text diagnosis also included pseudo, suspected, family history, or nephrolithiasis were also excluded. Finally, 98 subjects with the following diseases also known to affect sUA levels were excluded: Familial Mediterranean Fever (ICD-9 277.31) (*n* = 56), glycogen storage disease (ICD-9 271.0) (*n* = 42), Lesch-Nyhan syndrome (ICD-9 277.2) (*n* = 0), juvenile gout (ICD-9 277.2) (n = 0), tumor lysis syndrome (ICD-9 277.88) (n = 0), or lead toxicity associated with gout (ICD-9 984.9) (*n* = 1).

Gout patients were categorized by the presence of CKD at index date as defined by an indication of moderate/severe chronic kidney disease (based on laboratory values and using the CKD-EPI creatinine equation to identify CKD ≥ 3 [37], or a diagnosis or claim for chronic renal failure, kidney transplantation, or dialysis.

### Measures

Demographic variables such as age, sex were collected at index date. Age was assessed continuously and by groups < 55 and 55+ years as quality of gout healthcare management has been shown to decrease with older ages [13]. Socio-economic status (SES) (low, medium, high, or missing) is an area-level score calculated based on current or last place of residence thus it likely reflects the patient’s SES at the end of follow-up. Misclassification of SES as a result of this definition is considered minimal as SES is considered to be stable during the non-critical 5-year period of adulthood in comparison to potential shifts during one’s early life course [38]. In addition, the use of the latter SES indicator, as an adjustment for the confounding effect on resource utilization, is perhaps a better indicator of the cumulative influence of SES [39]. Individual-level SES data are not collected by any health plan in Israel due to Israeli law, therefore SES scores derived by the Israel Central Bureau of Statistics and based on small statistical areas were used [40, 41].

Comorbid conditions at or prior to index date include cancer (ICD-9 140–208), cardiovascular disease (CVD) (ICD-9 410, 411, 413, 414, 429–434, 436, 438, V45.81/2, and coronary artery stent insertion and aortic bypass surgery procedures), diabetes (ICD-9 250), and hypertension (ICD-9 401–405) were identified using CHS algorithms [36, 42]. The Charlson Comorbidity Index (CCI) [43], was used to represent a weighted sum of multiple comorbid conditions predictive of higher resource utilization. Greater scores indicated a greater comorbid burden on the patient.

Clinical characteristics included smoking habits (current smoker, former smoker, and never smoker) and body mass index (BMI) (continuous and categorical coded according to the World Health Organization as: underweight [< 18.5 kg/m^2^], normal weight [18.5 to < 25.0 kg/m^2^], overweight [25.0 to < 30.0 kg/m^2^], obese [≥ 30.0 kg/m^2^], or missing).

Healthcare resource utilization for the five follow-up years following index date was calculated as the mean of the total number per year of general practitioner visits, specialist visits (e.g., rheumatologist or orthopedist), hospital admissions, use of imaging services (x-ray, MRI, ultrasound, and CT), or allopurinol (ATC M04AA01) purchase similar to others’ methods [3, 19, 44]. Mean total number of tests and test values for sUA levels (last test value prior to index date) ≤ 6 or > 6 mg/dL were reported. Survival was examined using date of death.

### Statistical analysis

Age-adjusted incidence was calculated using the 2009 Clalit population distribution and direct standardization according to the Israeli population in 2009 (Central Bureau of Satistics, 2010) was used to calculate age-standardized incidence of gout [45]. Standardized rates and their 95% confidence intervals (CI) were used to assess age-standardized rate ratios by sex. Descriptive analyses were performed to characterize the patient population’s demographic, medical history, and clinical characteristics of patients at index date.

Generalized estimating equations for repeated measures were used to assess change in healthcare utilization over the 5-year follow-up period for gout patients with and without pre-existing CKD at index date and stratified by age groups < 55 and 55+ years. Model distributions differed depending on outcome variable (general practitioners visits, allopurinol purchase = normal; specialist visits, hospitalizations, and sUA testing = negative binomial; imaging [data were restructured to binomial data, i.e., yes/no annual testing, to account for correlated data due to multiple testing for a single event] = binomial) with a first order autoregressive (AR (1)) correlation structure. The AR (1) order is used since the model is fitting longitudinal repeated measures of correlated data and similar estimates were observed when using the unstructured correlation structure. Annual unit change and 95% CI were presented and when appropriate, data were transformed from the logarithmic scale. Data prior to index date was indexed as year 0 and all subsequent years as years 1 through 5. All models were adjusted for age, sex, smoking status (current vs non-current), SES (low vs other), and CCI. During the 5-year follow-up 1673 (20.2%) healthy gout patients developed CKD. Change in patient kidney disease status from index date was not adjusted for in models since the goal of the analysis was to examine utilization based on characteristics at index date.

Time-to-death was examined using Kaplan Meier survival curves and the log rank test were used to test equality of survival distributions between the subgroups with and without CKD and stratified by ages < 55 and 55+ years. Patients were right-censored according to the month they left the health plan. Cox proportional hazard models were used to assess the risk of death in patients with and without CKD at index date and stratified by age group < 55 and 55+ years accounting for age, sex, SES, CCI, smoking status, BMI, sUA control, and gout medication use. Proportional hazard assumptions were used examining the effect of age within each age group. Hazard ratios (HR) and 95% CI were reported.

Analyses were conducted using SPSS version 23.

## Results

**Fig. 2:**
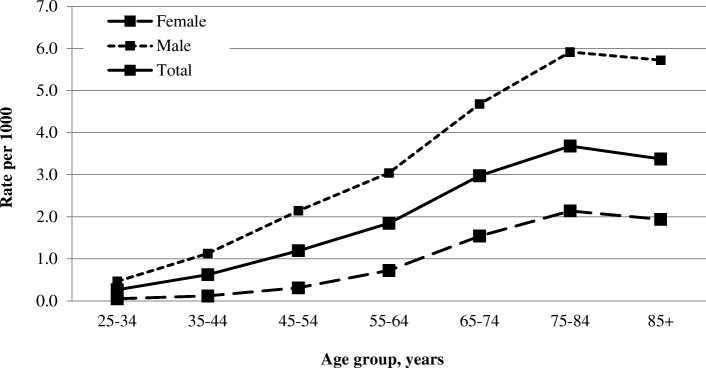
Cumulative incidence of gout by age and sex (2006–2009) (*n* = 12,940)

A total of 12,940 incident adult gout patients were included in the study. Age-specific cumulative incidence rates over a 4-year period per 1000 Clalit members increased for both sexes with age, reaching the highest rates for adults 75–84 years (overall = 3.68 per 1000; men = 5.91; women = 2.14) (Fig. 2). The age-standardized cumulative incidence for this 4-year time period is 1.28 per 1000 (95% CI 1.23–1.32). A higher cumulative incidence was observed for men (2.07 per 1000, 95% CI 1.99–2.16) compared to women (0.55 per 1000, 95% CI 0.52–0.59) with a standardized rate ratio of 3.74 (95% CI 3.45–4.05, *p* < 0.05).

During the 5 years of follow-up from index date, there were 8286 (64.0%) and 4654 (36.0%) gout patients with and without CKD, respectively (Table 1). Of this cohort, 3421 patients died (26.4%) and 233 (1.8%) left Clalit with a total of 55,206 person-years of follow-up. The average age of gout patients at index date was 63.8 ± 15.6 years. Thirty-six percent (*n* = 4654) of all incident cases of gout had CKD at their index date. Gout patients without CKD at index date tended to be younger (without CKD = 58.1 ± 14.7 years vs with CKD = 74.0 ± 11.3 years), male (without CKD = 80.1% vs with CKD = 68.4%), of low SES (without CKD = 19.3% vs with CKD = 16.5%), and current smokers (without CKD = 15.8% vs with CKD = 6.8%) compared to those with these conditions. The health status of gout patients at index date indicated that 43.0% of patients are obese, with a larger percentage among those without (45.3%) than with (38.8%) CKD at index date. CVD (38.4%), diabetes (28.9%), hypertension (65.6%) and cancer (12.1%) were the most common comorbidities in gout patients at index date and were substantially higher among those with CKD at index date. Similarly, upon diagnosis 69.1% of gout patients had a CCI ≥ 1 with higher CCI scores for those with compared to those without CKD at index date (without CKD = 58.0% vs with CKD = 89.0%).

**Table 1:** Characteristics at diagnosis of patients with incident gout between 2006 and 2009

	All gout patients	Gout patients without CKD	Gout patients with CKD	*p*-value^c^
Total	12,940	8286	4654	
Died during follow-up, n (%)	3421 (26.4%)	1110 (13.4%)	2311 (49.7%)	< 0.001
Person-years	57,206	39,014	18,192	
Age (years)				
Mean ± SD	63.8 ± 15.6	58.1 ± 14.7	74.0 ± 11.3	< 0.001
IQR 25–75	53–76	48–69	68–82	
Age, n (%)				< 0.001
25–34 years	621 (4.8%)	590 (7.1%)	31 (0.7%)	
35–44 years	992 (7.7%)	940 (11.3%)	52 (1.1%)	
45–54 years	1924 (14.9%)	1740 (21.0%)	184 (4.0%)	
55–64 years	2799 (21.6%)	2210 (26.7%)	589 (12.7%)	
65–74 years	2917 (22.5%)	1637 (19.8%)	1280 (27.5%)	
75–84 years	2739 (21.2%)	946 (11.4%)	1793 (38.5%)	
85+ years	948 (7.3%)	223 (2.7%)	725 (15.6%)	
Sex, n (%)				< 0.001
Male	9819 (75.9%)	6634 (80.1%)	3185 (68.4%)	
Female	3121 (24.1%)	1652 (19.9%)	1469 (31.6%)	
Socio-economic status, n (%)				< 0.001
Low	2495 (19.3%)	1728 (20.9%)	767 (16.5%)	
Medium	5717 (44.2%)	3582 (43.2%)	2135 (45.9%)	
High	4651 (35.9%)	2913 (35.1%)	1738 (37.3%)	
Missing	77 (0.6%)	63 (0.8%)	14 (0.3%)	
Smoking habits, n (%)				< 0.001
Never smoker	7652 (59.1%)	4818 (58.2%)	2834 (60.9%)	
Former smoker	3346 (25.9%)	2025 (24.4%)	1321 (28.4%)	
Current	1628 (12.6%)	1313 (15.8%)	315 (6.8%)	
Missing	314 (2.4%)	130 (1.6%)	184 (4.0%)	
BMI (kg/m^2^), n (%)				< 0.001
Underweight	60 (0.5%)	35 (0.4%)	25 (0.5%)	
Normal	2013 (15.6%)	1095 (13.2%)	918 (19.7%)	
Overweight	4794 (37.0%)	3097 (37.4%)	1697 (36.5%)	
Obese	5559 (43.0%)	3755 (45.3%)	1804 (38.8%)	
Missing	514 (4.0%)	304 (3.7%)	210 (4.5%)	
Comorbid condition at index date, n (%)				
CVD	4968 (38.4%)	2070 (25.0%)	2898 (62.3%)	< 0.001
Diabetes	3743 (28.9%)	1883 (22.7%)	1860 (40.0%)	< 0.001
Hypertension	8494 (65.6%)	4367 (52.7%)	4127 (88.7%)	< 0.001
Cancer	1571 (12.1%)	696 (8.4%)	875 (18.8%)	< 0.001
CCI				
Mean ± SD	2.1 ± 2.3	1.2 ± 1.5	3.6 ± 2.7	< 0.001
IQR 25–75	0–3	0–2	2–5	
CCI, n (%)				< 0.001
0	3987 (30.9%)	3473 (42.0%)	513 (11.0%)	
1	2985 (23.1%)	2376 (28.7%)	609 (13.1%)	
2	1853 (14.3%)	1168 (14.1%)	685 (14.7%)	
3	1300 (10.1%)	602 (7.3%)	698 (15.0%)	
4	906 (7.0%)	305 (3.7%)	601 (12.9%)	
5	648 (5.0%)	174 (2.1%)	474 (10.2%)	
6	477 (3.7%)	94 (1.1%)	383 (8.2%)	
7+	767 (5.9%)	78 (0.9%)	689 (14.8%)	
Missing	17 (0.1%)	16 (0.2%)	2 (0.0%)	
sUA levels (mg/dL)				
Mean ± SD	8.1 ± 2.0	7.6 ± 1.7	8.9 ± 2.1	< 0.001
IQR25–75	6.8–9.3	6.4–8.7	7.5–10.1	
sUA levels (mg/dL)				< 0.001
≤ 6	1343 (10.4%)	981 (11.8%)	362 (7.8%)	
> 6	7796 (60.2%)	4175 (50.4%)	3621 (77.8%)	
Missing	3801 (29.4%)	3130 (37.8%)	671 (14.4%)	
Gout medication^b^, n (%)	4068 (31.4%)	2245 (27.1%)	1823 (39.2%)	< 0.001

Note: BMI = body mass index; BP = blood pressure; CCI=Charlson Comorbidity Index: CKD = chronic kidney disease; CVD = cardiovascular disease; IQR = interquartile range; SD = standard deviation; sUA = serum uric acid

^a^Values for most clinical characteristics at index date are the last value in the year prior to index date. In cases where this value is missing the following were used: smoking – closest record to index date through follow-up; BMI – closest record to index date for up to 3 years follow-up; systolic BP, diastolic BP, glucose, and creatinine – closest record to index date for up to 1 year follow-up. sUA levels were the lowest level during the 12 months prior to index date. Patients were considered positive for CVD, diabetes or hypertension if they ever received a diagnosis prior to index date, and positive for diuretic use if they purchased a diuretic in the year prior to index date. The CCI was the value at index date, 17 cases had missing values

^b^Gout medication is defined as any purchase of at least one of the following medications prior to index date: allopurinol, febuxostat, probenecid, sulfinpyrazone, or colchicine

^c^The chi square test, ANOVA or t-test were used to assess differences in distribution between the CKD groups

Prior to index date, 29.4% of patients had no recorded sUA level. Patients without compared to those with CKD at index date had substantially higher missing test values (37.8% versus 14.4%). Of those with available sUA test data, 19.0% of patient without CKD had controlled sUA (< 6 mg/dl) at index date compared to 9.1% of patients with CKD at index date. Mean sUA levels were 7.6 ± 1.7 and 8.9 ± 2.1 mg/dL for those without and with CKD, respectively (*p* < 0.001). In addition, at index date 31.4% of patients had purchased at least one gout-related medication in the previous year, with higher rates among those with compared to without CKD at index date (with CKD = 27.1% vs without CKD = 39.2%).

Healthcare utilization was calculated from one-year prior to index date to each year after index date over the 5-year follow-up period for patients with and without CKD and stratified by age group < 55 and 55+ years according to the population at the beginning of each follow-up year (see Table 2). Over the 5-year follow-up period from index date, 33.6% of patients presenting with CKD at index date either died or left Clalit, compared to 9.3% of those without CKD at cohort entry. Substantially more patients were lost to follow-up among the older age group (12.4% without CKD and 35.3% with CKD) compared to gout patients in the younger age group (4.4% without CKD and 8.5% with CKD). During the year prior to diagnosis, younger patients with CKD compared to patients without CKD had twice as many general practitioner visits and imaging tests performed, over four times more hospital admissions, almost triple the average months of allopurinol purchases per year and the number of sUA tests performed. Similar, but more attenuated differences were observed for those in the older age group. Between group differences for patients with versus without CKD were observed for unadjusted and adjusted models (*p* < 0.001). There were statistically significant changes in rates from year 1 to year 5 for healthcare utilization among all gout patients (*p* < 0.05) with the exception of younger patients and general practitioner visits where rates remained stable over time regardless of CKD status (Table 2). Among younger patients, the rate of change over time did not differ for those with or without CKD. Decreasing number of visits per patient were observed for specialists, imaging, hospitalizations and sUA testing, while the number of months of allopurinol use increased similarly for those with and without CKD over time. For example, those with CKD < 55 years purchased allopurinol on average for 3.49 ± 4.15 months in their first year from diagnosis and 4.36 ± 4.61 months in their fifth year from diagnosis. Among those 55+ years, the rate of change differed significantly between those with and without CKD for (*p* < 0.05) for average number per year of general practitioner visits, specialist visits, and months of allopurinol purchases. Healthcare resource use decreased for all with the exception of monthly allopurinol purchase for CKD patients 55+ years where rates increased from 2.71 ± 4.05 to 3.39 ± 4.63 from year 1 to 5.

**Table 2:** Average annual healthcare utilization among gout patients with and without CKD by age group

	At diagnosis^c^	Year from diagnosis^d^					Unit Change in Healthcare Utilization Per Year (95% CI)^e,f^
		1	2	3	4	5	
AGE 25–54 YEARS							
Number of patients	3537	3537	3498	3443	3408	3370	
% Follow-up without CKD	3272	100.0%	98.9%	97.6%	96.6%	95.6%	
% Follow-up with CKD	265	100.0%	98.9%	94.7%	93.8%	91.5%	
Average annual total number							
General healthcare utilization							
General practitioner visits							
Without CKD	7.1 ± 8.0	10.3 ± 9.5	9.6 ± 9.1	10.0 ± 9.2	10.3 ± 9.2	10.5 ± 9.3	0.03 (− 0.5–0.10)
With CKD	15.0 ± 11.6	18.3 ± 14.1	17.1 ± 12.7	17.5 ± 13.7	17.4 ± 14.2	17.7 ± 13.7	
Specialist visits^a^							
Without CKD	0.51 ± 1.25	0.83 ± 1.63	0.60 ± 1.57	0.59 ± 1.38	0.58 ± 1.40	0.58 ± 1.41	−0.09 (− 0.11– − 0.07)
With CKD	0.67 ± 1.44	0.80 ± 1.42	0.52 ± 1.26	0.55 ± 1.12	0.50 ± 1.14	0.43 ± 0.93	
Hospital admissions							
Without CKD	0.13 ± 0.55	0.24 ± 0.75	0.19 ± 0.72	0.18 ± 0.74	0.19 ± 0.67	0.19 ± 0.67	−0.05 (− 0.08– − 0.02)
With CKD	0.51 ± 1.21	0.81 ± 1.82	0.70 ± 1.45	0.60 ± 1.33	0.58 ± 1.26	0.57 ± 1.33	
Imaging use^b^							
Without CKD	1.03 ± 1.97	1.23 ± 2.24	0.95 ± 2.08	0.93 ± 2.04	0.95 ± 2.12	0.95 ± 2.09	−0.08 (− 0.10– − 0.07)
With CKD	1.99 ± 3.33	2.63 ± 4.25	1.93 ± 3.92	1.55 ± 3.15	1.52 ± 2.99	2.07 ± 4.36	
Gout-related healthcare utilization							
Allopurinol purchase months							
Without CKD	0.40 ± 1.59	1.64 ± 3.07	1.64 ± 3.22	1.85 ± 3.42	1.94 ± 3.51	2.17 ± 3.70	0.13 (0.10–0.16)
With CKD	1.14 ± 2.68	3.49 ± 4.15	3.67 ± 4.24	3.92 ± 4.27	4.00 ± 4.39	4.36 ± 4.61	
Serum uric acid testing							
Without CKD	0.91 ± 1.24	1.49 ± 1.59	1.04 ± 1.46	1.10 ± 2.25	1.16 ± 3.84	1.18 ± 2.88	−0.04 (−0.06– −0.02)
With CKD	3.20 ± 4.70	4.02 ± 7.15	3.64 ± 6.32	3.79 ± 8.98	3.41 ± 5.76	3.95 ± 7.58	
AGE 55+ YEARS							
Number of patients	9403	9403	8674	8181	7675	7231	
% Follow-up without CKD	5012	100.0%	96.1%	93.4%	90.2%	87.6%	
% Follow-up with CKD	4391	100.0%	87.9%	79.7%	71.8%	64.7%	
Average annual total number							
General healthcare utilization							
General practitioner visits							
Without CKD	12.8 ± 11.3	16.1 ± 12.4	15.9 ± 12.2	16.3 ± 12.4	16.6 ± 12.7	16.5 ± 12.6	−0.79 (−0.88 – −0.70)
With CKD	18.4 ± 14.5	21.1 ± 15.5	20.8 ± 14.8	21.0 ± 15.2	20.8 ± 15.0	20.6 ± 15.2	−0.34 (− 0.48 – − 0.20)
Specialist visits^a^							
Without CKD	0.72 ± 1.50	0.88 ± 1.64	0.73 ± 1.50	0.71 ± 1.49	0.69 ± 1.44	0.64 ± 1.34	−0.04 (− 0.05 – − 0.03)
With CKD	0.69 ± 1.47	0.82 ± 1.58	0.67 ± 1.40	0.65 ± 1.42	0.63 ± 1.39	0.59 ± 1.33	−0.11 (− 0.13 – − 0.09)
Hospital admissions							
Without CKD	0.29 ± 0.78	0.58 ± 1.23	0.44 ± 1.04	0.44 ± 1.03	0.43 ± 1.08	0.43 ± 1.04	−0.03 (− 0.04 – − 0.02)
With CKD	0.46 ± 1.11	0.84 ± 1.55	0.64 ± 1.33	0.63 ± 1.35	0.62 ± 1.37	0.61 ± 1.33	
Imaging use^b^							
Without CKD	1.71 ± 2.95	2.14 ± 3.40	1.70 ± 3.06	1.57 ± 2.82	1.50 ± 2.82	1.52 ± 2.84	−0.07 (− 0.09 – − 0.06)
With CKD	2.01 ± 3.30	2.46 ± 3.93	1.86 ± 3.38	1.67 ± 3.17	1.54 ± 3.03	1.45 ± 2.93	
Gout-related healthcare utilization							
Allopurinol purchase months							
Without CKD	0.79 ± 2.49	2.20 ± 3.77	2.33 ± 3.97	2.51 ± 4.11	2.75 ± 4.26	2.97 ± 4.48	−0.15 (−0.17 – − 0.12)
With CKD	0.98 ± 2.72	2.71 ± 4.05	2.86 ± 4.26	3.02 ± 4.35	3.19 ± 4.43	3.39 ± 4.63	0.03 (−0.03–0.05)
Serum uric acid testing							
Without CKD	1.37 ± 2.02	2.11 ± 3.50	1.76 ± 3.64	1.68 ± 2.36	1.73 ± 2.59	1.80 ± 2.80	−0.03 (− 0.04– − 0.02)
With CKD	2.02 ± 2.94	2.86 ± 4.23	2.42 ± 4.07	2.31 ± 3.46	2.32 ± 3.54	2.34 ± 3.69	

Note: CKD = chronic kidney disease

^a^Specialists include rheumatologist or orthopedist

^b^Imaging use includes x-ray, CT, ultrasound or MRI

^c^Data were collected during the year prior to diagnosis

^d^Group differences were significant for all models at *p* < 0.001

^e^A single rate of change is presented when there is no statistically significant difference in change over time by group

^f^All changes in rates from year 1 to year 5 were statistically significant at *p* < 0.05 with the exception of patients 25–54 years and general practitioner visits

**Fig. 3:**
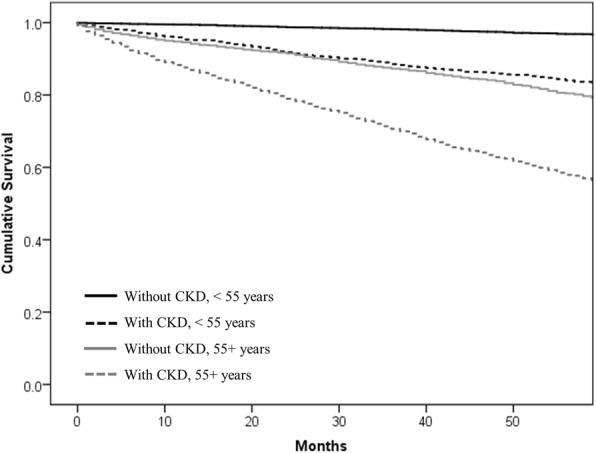
Kaplan-Meier curve for five-year survival among gout patients with and without CKD by age group

Survival curves were estimated for this patient population illustrating significant differences in survival probabilities by subgroups, CKD status and age group (*p* < 0.001) (Fig. 3). Of the 3421 patients who died during the 5-year study period, the majority had CKD (without CKD < 55 years = 4.9%; with CKD < 55 years = 22.4%; without CKD 55+ years = 29.9%; with CKD 55+ years =55.8%). Mean survival times differed significantly between groups (without CKD < 55 years: 59.1 ± 6.0 months, with CKD < 55 years: 54.5 ± 14.3 months, without CKD 55+ years: 53.5 ± 15.3 months, with CKD 55+ years: 45.4 ± 20.3 months; *p* < 0.001). Cox regression models assessed hazard of survival from diagnosis and adjusted for index date characteristics: age, sex, SES, CCI, smoking status, BMI, sUA control (< or ≥ 6 mg/dl), and gout medication use. Cox models were examined for those < 55 and 55+ years. For both age groups, the risk of dying was higher among those with compared to those without CKD (HR_< 55 years_ = 1.65; 95% CI 1.01–2.71; HR_55+ years_ = 1.50; 95% CI 1.37–1.65).

## Discussion

The present population-based study followed 12,940 incident gout patients for 5 years and compared healthcare utilization for patients with CKD and without CKD at their gout diagnosis. We demonstrated that patients with gout and CKD have greater rates of healthcare utilization at the start of follow-up than those without CKD, regardless of age. The additional health impairment of the patient with gout and CKD did not affect the rate of change on the burden of healthcare utilization over the 5-year period for adults < 55 years, however differentially influenced the rate of healthcare resource utilization (HRU) use for patients 55+ years. An increased mortality risk of 65 and 71% for those with CKD compared to without CKD was observed for patients < 55 years and 55+ years, respectively. The expected differences in HRU among those with CKD and elderly compared to non-CKD and younger groups, were not unexpected, however were not consistently apparent across all resource types and groups.

The relationship between gout and kidney disease is complex. Patients with gout are at an increased risk of CKD and CKD-related mortality, and patients with CKD are at an increased risk for hyperuricemia, which is a risk factor for gout [21, 46, 47]. For all gout patients, recommendations support the ability and need to control sUA levels to < 6 mg/dl [8, 48]. Reaching this target requires close monitoring due to potential drug-drug interactions and adverse events related to ULT use especially for CKD patients [48, 49]. For example, treating gout patients with reduced renal function may require dose escalation of allopurinol to offset its interaction with furosemide [24, 48]. Among CKD patients, control of hyperuricemia is debated among experts [26, 48, 50] and clinical guidelines for these patients note that there is insufficient evidence to recommend lowering serum uric acid in order to prevent CKD progression [51, 52].

In the present study there were several indicators of hyperuricemia monitoring and control, including sUA testing rates, sUA levels prior to diagnosis, and allopurinol purchase. We observed consistently higher average sUA testing per year for patients with CKD compared to those without CKD, regardless of age suggestive of closer monitoring for this sub-group, however, change in rates over time did not reveal differential changes by these sub-groups. After controlling for confounding factors, the average number of months that a patient purchased allopurinol increased for younger patients irrespective of CKD status, decreased for older patients without CKD, and remained stable for older patients with CKD, indicating a sensitivity of approach to treating gout according to age and comorbid conditions. It should be noted that while uncontrolled disease and poor clinical outcomes are often attributed to inadequate clinical monitoring, patients in the current study had high annual testing rates and ever-tested rates in the previous 5-year period relative to the reported rates in other countries [11, 19]. Finally, while the relationship between sUA monitoring over time and mortality was not assessed, we saw substantially higher mortality rates over the follow-up period for those with than those without CKD. These results may thus provide important insight into the burden of this comorbid disease, which persists despite effective clinical monitoring.

The ability to identify incident cases of gout, classify them according to CKD status and follow their healthcare utilization and survival over a 5-year period is a strength of this study and adds further perspective to the complexities related to gout management. The healthcare system in Israel is universal and provides physician care and a basic basket of medications and services to all residents. Conducting a population-based study utilizing data from the largest health care payer/provider in Israel provides an opportunity to study healthcare utilization and survival in gout patients independent of the effect of access to care. The relevance of these findings lies in the generalizability of the Israel cohort to that of others. Specifically, it is important to note that the incidence of gout in Israel of 1.29 per 1000 (95% CI 1.23–1.32) and rates by sex are similar to those reported in other countries, such as Sweden, Taiwan and UK [20, 53, 54]. Likewise, socio-demographic characteristics of the cohort are similar to that of other patient cohorts, with higher proportion of gout cases among older adults and men, and an unclear association with SES [19, 54, 55].

The results, however, are not without their limitations. First, clinical notes were not available for analysis in the dataset, which may lead to a misinterpretation of a healthcare encounter. Second, since we did not account for change in CKD status from index date, our results may reflect an attenuated relationship. Specifically, if those in the non-CKD group develop CKD during the follow-up period, their HRU and survival will be more similar to the CKD group. Also, the use and dose of ULT may not be equally distributed among CKD and age groups and may influence disease control and the resultant resource use. The potential bias associated with this uncontrolled confounder is unknown, however, others using data from Israel showed that medication adherence is related to SES, which is controlled for in the regression models and that those with comorbidities have improved compliance [30]. Next, identification of incident cases was limited by the availability of historical data by which we assumed the index date to be the first encounter for gout with the EHR system and not the first diagnosis. The Clalit EHR records are incomplete with regards to emergency department use due to out of system use. The lack of this information detracts from assessing the full extent of gout, regardless of subgroup on the economic burden to the healthcare system. Further insights into the relationships identified in the study were limited by our inability to examine time from disease diagnosis, gout flares or tophi, and cause of death. In addition, while the relatively large sample size allowed for increased power to detect difference, caution should be applied to the relevance of difference.

## Conclusions

In light of the challenges facing clinicians to ‘cure’ gout, these findings provide critical evidence of differences between patient characteristics, healthcare utilization and outcomes of this at-risk sub-group over the course of the disease. Future work should explore factors associated with these outcomes and barriers to gout control such as annual sUA testing and medication adherence, to better understand patient management by these subgroups.

## Abbreviations

ACR: American College of Rheumatology
AR(1): First order autoregressive
BMI: Body mass index
CCI: Charlson Comorbidity Index
CHS: Clait Health Services
CI: Confidence interval
CKD: Chronic kidney disease
CVD: Cardiovascular disease
EHR: Electronic health record
EULAR: European League Against Rheumatism
HR: Hazard ratio
HRU: Healthcare resource utilization
ICD-9: International Classification of Diseases 9th version
IQR: Interquartile range
NHANES: National Health and Nutrition Examination Surveys
SD: Standard deviation
SES: Socio-economic status
sUA: Serum uric acid
ULT: Urate-lowering therapy
